# Leaching of soils during laboratory incubations does not affect soil organic carbon mineralisation but solubilisation

**DOI:** 10.1371/journal.pone.0174725

**Published:** 2017-04-05

**Authors:** Beatriz González-Domínguez, Mirjam S. Studer, Frank Hagedorn, Pascal A. Niklaus, Samuel Abiven

**Affiliations:** 1 Soil Science and Biogeochemistry, Department of Geography, University of Zurich, Zurich, Switzerland; 2 Department of Evolutionary Biology and Environmental Studies, University of Zurich, Zurich, Switzerland; 3 Forest Soils and Biogeochemistry, Swiss Federal Institute for Snow, Forest and Landscape Research WSL, Birmensdorf, Switzerland; Pacific Northwest National Laboratory, UNITED STATES

## Abstract

Laboratory soil incubations provide controlled conditions to investigate carbon and nutrient dynamics; however, they are not free of artefacts. As carbon and nitrogen cycles are tightly linked, we aimed at investigating whether the incubation-induced accumulation of mineral nitrogen (N_min_) biases soil organic carbon (SOC) mineralisation. For this, we selected two soils representative of the C:N ratio values found in European temperate forests, and applied two incubation systems: ‘closed’ beakers and ‘open’ microlysimeters. The latter allowed leaching the soil samples during the incubation. By the end of the 121-day experiment, the low C:N soil significantly accumulated more N_min_ in beakers (5.12 g kg^-1^ OC) than in microlysimeters (3.00 g kg^-1^ OC) but there was not a significant difference in SOC mineralisation at any point of the experiment. On the other hand, N_min_ did not accumulate in the high C:N soil but, by the end of the experiment, leaching had promoted 33.9% more SOC solubilisation than beakers. Therefore, we did not find evidence that incubation experiments introduce a bias on SOC mineralisation. This outcome strengthens results from soil incubation studies.

## Introduction

Laboratory soil incubations have been extensively applied in various areas of research [1,2] and are commonly used to investigate the potential mineralisation of soil organic carbon (SOC) under optimum conditions for microbial activity [3]. Compared to field experiments, laboratory incubations have the advantages of standardising soil samples (e.g. particle size, bulk density) and controlling environmental drivers of mineralisation (e.g. temperature, moisture content). During the decomposition of soil organic matter, ammonification transforms organic nitrogen into NH_4_^+^, which is transformed into NO_2_^-^ and NO_3_^-^ through nitrification. Ammonification is part of the decomposition process of soil organic matter and leads to a net production of CO_2_, even though the chemoautotrophic oxidation of NH_4_^+^ requires CO_2_. In the field, mineral nitrogen (N_min_) is susceptible to being leached down the profile. However, in beakers, the ‘closed’ and most frequently used incubation system [4], this removal does not occur. As a result, N_min_ may accumulate in the soil sample potentially affecting SOC mineralisation. In an attempt to minimise this incubation-induced accumulation of N_min_, in this study we applied an ‘open’ incubation system, referred to as microlysimeter [5–7], that permits the leaching of soil samples during the experiment.

Most incubation studies with N-amended soils apply the treatment at the beginning of the experiment and have shown that increases in N_min_ concentrations typically decrease native SOC mineralisation [8,9]. Potential reasons for this decrease are the decline in N-mining by soil microbial communities [10] and the suppression of extracellular enzymes [11]. However, there are also incubation studies that have shown no effect [12] or a positive effect of N_min_ on SOC mineralisation in cases of soils with severe N limitation [13]. As the availability of N_min_ is influential on substrate decomposition [14], bulk soil C:N ratios can contribute to explain the dynamics of these two elements in soils.

In this study, we compared ‘closed’ (i.e beakers) and ‘open’ (i.e. microlysimeters) incubation systems and tested whether N_min_ accumulation occurred and whether it had an effect on SOC mineralisation. We hypothesise that the effect of the incubation-induced accumulation of N_min_ on SOC mineralisation depends on the C:N ratio of the soil, with suppressed and stimulated mineralisation in low and high C:N soils respectively. This hypothesis is of particular relevance to laboratory incubation studies. If proved true, C mineralisation measured in these experiments could be differently biased depending on the C:N ratio of the incubated sample.

## Materials and methods

### Ethics statement

Sites from where soils for this study were sampled are part of the Swiss Federal Institute of Forest, Snow and Landscape Research network. All necessary permits were obtained before sampling. This study did not involve endangered or protected species.

### Selection, sampling and characterization of study soils

To test our hypothesis, we selected two soils (Table 1) from the database of the Swiss Federal Institute for Forest, Snow and Landscape Research (WSL) [15]. In May 2014, this database contained data on 1,050 soil profiles spread across Switzerland. The C:N ratio of the two soils selected (12.51 ± 0.03 and 17.43 ± 0.17, mean ± s.e.m.) was representative of the values most commonly found in European temperate forest mineral soils [16]. But at the same time, these two soils were different within the range of probability distribution of C:N values in Swiss forest soils (2^nd^ and 7^th^ decile); therefore, representing soils of contrasting N_min_ dynamics.

**Table 1:** Characteristics of the two mineral forest soils (upper 20 cm) used in this study.

	Low C:N soil	High C:N soil
Coordinates (WGS84)	46.680°N, 6.898°E	46.268°N, 7.436°E
Forest type	Beech	Pine
Soil type	Phaeozem	Calcisol
Soil C:N ratio	12.51 ± 0.03	17.43 ± 0.17
Soil total organic C (g kg^-1^)	36.70 ± 2.15	36.63 ± 3.81
Soil total N (g kg^-1^)	2.93 ± 0.17	2.10 ± 0.21
Soil pH (CaCl_2_)	6.74 ± 0.02	5.83 ± 0.06
Clay (%)	24.36	15.83
Texture class (USDA)	Loamy	Silty-loamy
Fe (NH_4_Cl extraction, mmol_c_ kg^-1^)	0.0044	0.0037
Al (NH_4_Cl extraction, mmol_c_ kg^-1^)	0.0000	0.0235

In August 2014, we collected three soil composites within a 40 × 40 m^2^ plot at each site. Each composite was the product of mixing eight 0–20 cm depth soil cores collected from one of three non-overlapping areas of the plot. This sampling strategy enabled us to account for spatial variability. Each composite constituted an experimental replicate. Soil samples were collected with a 5 cm diameter Humax corer. After collection, samples were transported in portable fridges to the lab where they were freshly sieved by hand (≤ 2 mm) and stored at 3.5°C until the beginning of the experiment in March 2015.

We measured soil pH on 40°C dried composite subsamples. Part of these dried soil subsamples were also milled and fumigated with HCl to measure total organic carbon and total nitrogen with an Elemental Analyser (vario MICRO cube, Elementar, Germany). The rest of data in Table 1 is part of the WSL database [15,17].

### Incubation experiment

We incubated fresh soil (sieved to ≤ 2 mm; 40 g equivalent dry mass; adjusted to 0.8 g cm^-3^ bulk density) in sterilised glass beakers and microlysimeters. Soil samples in both incubation systems were placed separately into 2 litre airtight glass jars that contained 20 ml of distilled water to ensure the headspace was moist. Lasting 121 days, the experiment was conducted under aerobic conditions, in the dark and at 25°C. The experiment started after a 10-day pre-incubation which was run under the same conditions as in the incubation assay.

During the course of the experiment, we repeatedly measured SOC mineralisation and the concentration of N_min_ and organic carbon (OC) in two types of soil water extracts (SWE). The CO_2_-C product of the mineralisation of SOC was captured in 20 ml NaOH (1M) traps placed into the 2 liter glass jars. Subsequently, the amount of C trapped was determined by the change of conductivity of the NaOH [18]. Mineral N (NO_3_^-^ and NO_2_^-^) in SWE was quantified with a continuous flow analyser (San++, Skalar, Breda, the Netherlands). We also measured NH_4_^+^ but the concentrations were below the detection limit (1 ppm) and are not reported. Finally, OC in SWE was determined with a TOC analyser (DIMA TOC-2000 Dimatec, Essen, Germany).

Since the beginning of the experiment, NaOH traps were taken out and replaced for fresh ones on days 4, 13, 30, 63 and 121. On these five days, microlysimeters were leached (SWE_l_) and beakers and microlysimeters were destructively extracted with water (SWE_e_); hence we obtained two types of SWE samples. To produce SWE_l_ samples, 30 ml of nutrient solution [19] without N or P were added to each microlysimeter. Subsequently, after equilibration for 30 minutes, the systems were leached by applying a suction of -20 kPa for 25 minutes. To obtain SWE_e_ samples, three replicates of each combination of soil and incubation system (n = 12) were extracted with distilled water (1:5 soil:water) after shaking for 30 minutes at 100 rpm. SWE_l_ and SWE_e_ samples were filtered (1.6 μm MGA glass microfibre, Sartorius) before analysis of N_min_ and OC.

Until the time of a given leaching date, the total quantity of N_min_ or OC solubilised ([*SWE*_*T*_]_*t*_) for samples in microlysimeters, was the sum of the quantity of N_min_ or OC found in the SWE of the destructed sample ([*SWE*_*e*_]_*t*_), plus the cumulative quantity of N_min_ or OC removed in previous leaching cycles ($\sum_{i=1}^{t-1}{\left[{SWE}_{l}\right]}_{i}$) (Eq 1).

$${\left[{SWE}_{T}\right]}_{t}={\left[{SWE}_{e}\right]}_{t}+\sum_{i=1}^{t-1}{\left[{SWE}_{l}\right]}_{i}$$ (Eq 1)

Eq 1 does not apply to samples incubated in beakers. As ‘closed’ systems were not flushed, their total quantity of N_min_ or OC solubilised is equal to SWE_e_.

### Data analysis

Statistical analysis was performed with the R software (version 3.3.2) [20]. Incubation data (i.e. SWE_e_-N_min_, SWE_e_-OC, OC mineralised) were normalised relative to the total OC content of the bulk soil at the beginning of the experiment. Incubation systems and soil types were compared by Student’s *t*-tests. Results can be found in the Supplementary Material section. Errors given in the text, tables and graphs are standard errors of the mean. All data produced in this study is open access [21].

## Results

### Soluble mineral nitrogen

**Fig 1:**
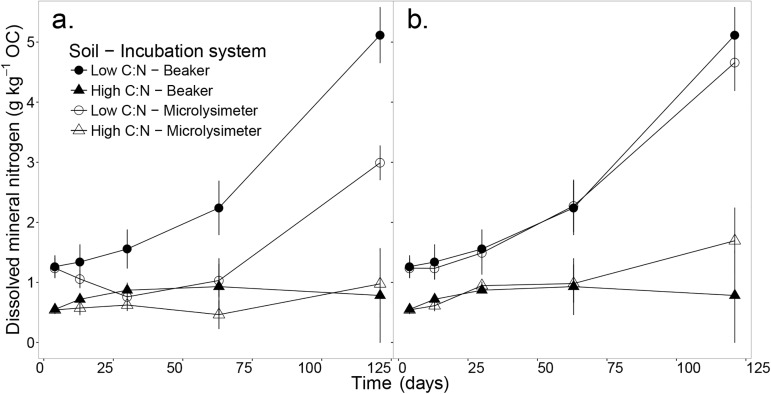
Soluble mineral nitrogen (NO_3_^-^-N and NO_2_^-^-N) relative to total soil organic carbon (OC). Soluble mineral nitrogen (NO_3_^-^-N and NO_2_^-^-N) relative to total soil organic carbon (OC) over a 121-day incubation. (a) Measured in 1:5 soil water extracts (*SWE*_*e*_) and (b) calculated (Eq 1) for the total soil water extract as the sum of extracted and leached N_min_ (*SWE*_*T*_), for two soils (i.e. high and low C:N ratios) and two incubation systems (i.e. leached microlysimeters and un-leached beakers). Error bars represent the standard error of the mean (n = 3).

Results show that the concentration of soluble N_min_ in the incubated samples related to the C:N ratio of the soil (Fig 1A, S1 Table). In both incubation systems, the low C:N soil accumulated N_min_ non-linearly over the incubation period, but by day 121 this accumulation was significantly smaller in microlysimeters compared to beakers (*p* < 0.05, *t*-test). In the high C:N soil, N_min_ remained constant in both incubation systems (Fig 1A). When examining the total N_min_ produced (Fig 1B, S1 Table), there was no significant difference between beakers and microlysimeters for any of the two soils.

### Soluble organic carbon

**Fig 2:**
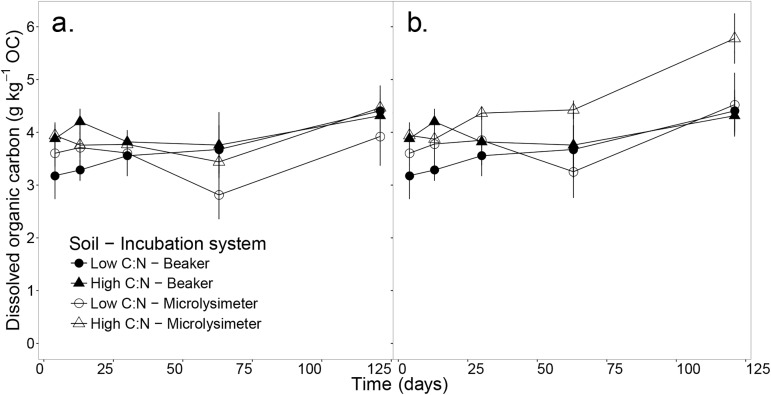
Soluble organic carbon relative to total soil organic carbon (OC). Soluble organic carbon relative to total soil organic carbon (OC) over a 121-day incubation. (a) Measured in 1:5 soil water extracts (*SWE*_*e*_) and (b) calculated (Eq 1) for the total soil water extract as the sum of extracted and leached OC (*SWE*_*T*_), for two soils (i.e. high and low C:N ratios) and two incubation systems (i.e. leached microlysimeters and un-leached beakers). Error bars represent the standard error of the mean (n = 3).

Soluble OC did not accumulate in any soil or incubation system (Fig 2A, S2 Table). However, when considering the total soluble OC produced over the incubation period (Fig 2B, S2 Table), we observe that by day 121, the high C:N soil released more OC in microlysimeters than in beakers (*p* = 0.07, *t*-test). Additionally, the cumulative OC leached from microlysimeters represented about 0.96 ± 0.13% and 1.79 ± 0.10% of the cumulative CO_2_-C released, in the low and high C:N soil respectively.

### Soil organic carbon mineralisation

**Fig 3:**
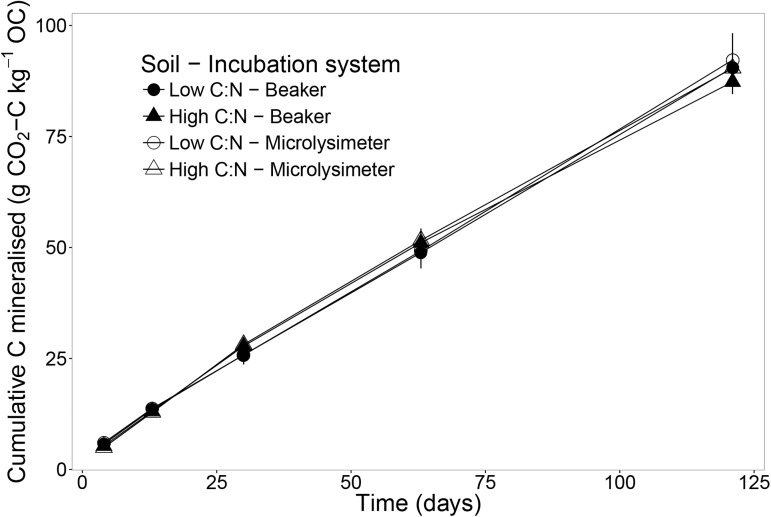
Cumulative carbon mineralised (CO_2_-C) relative to total soil organic carbon (OC). Cumulative carbon mineralised (CO_2_-C) relative to total soil organic carbon (OC) of two soils (i.e. high and low C:N ratios), incubated in two incubation systems (i.e. leached microlysimeters and un-leached beakers). Error bars represent the standard error of the mean (n = 3).

By day 121, there was no significant difference in the cumulative C mineralised between soils or incubation systems (Fig 3, S3 Table). Quantitatively, the C mineralised by the low C:N soil was 90.52 ± 4.00 g kg^-1^ OC in beakers and 92.23 ± 5.89 g kg^-1^ OC in microlysimeters. The high C:N soil mineralised 87.30 ± 2.62 g kg^-1^ OC in beakers and 90.39 ± 1.80 g kg^-1^ OC in microlysimeters.

## Discussion

### ‘Closed’ systems promoted the accumulation of N_min_ in the low C:N soil, but this accumulation did not significantly affect SOC mineralisation

Although we measured an increase of 2 and 3 times the initial N_min_ concentration in microlysimeters and beakers respectively by the end of the incubation of the low C:N soil (Fig 1A), we did not observe a significant effect of these two levels of N_min_ on SOC mineralisation (Fig 3). Therefore, this result contradicts our initial hypothesis, even considering that the increase of N_min_ in this soil in beakers (140.27 ± 3.70 μg N_min_ g^-1^ soil) was the same magnitude as than treatments applied in N-amended incubations and where a negative effect on SOC mineralisation was found [9]. One explanation for this result could be that the concentrations of N_min_ were not high enough to inhibit decomposition enzymes [22]. A second explanation could be that N-amended incubations are commonly short-term assays (i.e. hours, days) that apply a unique treatment at the beginning of the experiment [9,12,13,23]. Contrary to these experiments, the accumulation of N_min_ in this study was gradual and hence, soil microorganisms may have progressively adapted to the increasing N_min_ concentrations [23].

### Mineral N did not accumulate in the high C:N soil and leaching did not induce N limitation

In the high C:N soil in beakers, we observed a constant concentration of N_min_ (Fig 1A). This was probably due to a steady state between the production and the immobilisation of N_min_ by soil microorganisms [24,25]. Leaching did not induce N_min_ limitation in the high C:N soil. This conclusion is supported by the fact that the total N_min_ produced over the incubation period (Fig 1B) was not significantly larger in microlysimeter than in beakers.

### Leaching promoted the solubilisation of SOM in the high C:N soil

Leached OC represented on average only ~1% of the SOC mineralised as CO_2_; therefore, it is unlikely that the depletion of labile C from leaching had a significant effect on the mineralisation of SOC. We did not observe an accumulation of soluble OC over the incubation period in any soil or incubation system, but a stable concentration within the range of 2.8–4.5 g kg^-1^ OC (Fig 2A). This dynamic reflects an equilibrium between the solid and the aqueous phases of soil organic matter [24–28]. This explanation is also supported by the higher solubilisation of SOC in the leached high C:N soil compared to the un-leached system (Fig 2B).

## Conclusions

In this study, we aimed at investigating whether the incubation-induced accumulation of N_min_ biases SOC mineralisation. We selected two soils that (i) were representative of the C:N ratio values found in European temperate forests, and (ii) differed on their net nitrogen mineralisation. Results demonstrated that the progressive accumulation of N_min_, which only occurred in the low C:N soil, did not have a significant effect on the mineralisation of SOC. In parallel, N_min_ did not accumulate in the high C:N soil, but leaching promoted higher solubilisation of SOC. Our results are based on two representative European temperate forest soils, but incubations are applied to a wider range of soil types. Therefore, to test whether the results of this study hold independently of the characteristics of the incubated samples (e.g. pH, texture), future work should be undertaken with a broader range of soils, also including strongly N-limited ones.

## Supporting information

S1 Table Results of Student’s *t-*tests to compare two incubation systems (i.e. leached microlysimeters and un-leached beakers) in two soils (i.e. high and low C:N ratios). Data tested are soluble mineral nitrogen (NO_3_^-^-N and NO_2_^-^-N) relative to total soil organic carbon (Fig 1A and 1B) over the 121-day incubation (g kg^-1^ OC). SWE_e_-N_min_ was measured in 1:5 soil water extracts (Fig 1A) and SWE_T_-N_min_ was calculated (Eq 1) for the total soil water extract as the sum of extracted and leached N_min_ (Fig 1B). Cell values: Significance code based on *p-*values (‘‘1, ‘.’0.1, ‘*’0.05, ‘**’0.01, ‘***’0.001), *t-*value, p = *p*-value.
(DOCX)

S2 Table Results of Student’s *t-*tests to compare two incubation systems (i.e. leached microlysimeters and un-leached beakers) in two soils (i.e. high and low C:N ratios). Data tested are soluble organic carbon (OC) relative to total soil organic carbon (Fig 1A and 1B) over the 121-day incubation (g kg^-1^ OC). SWE_e_-OC was measured in 1:5 soil water extracts (Fig 2A) and SWE_T_-OC was calculated (Eq 1) for the total soil water extract as the sum of extracted and leached OC (Fig 2B). Cell values: Significance code based on *p-*values (‘‘1, ‘.’0.1, ‘*’0.05, ‘**’0.01, ‘***’0.001), *t-*value, p = *p*-value.
(DOCX)

S3 Table Results of Student’s *t-*tests to compare: in *row-1*, two incubation systems (i.e. leached microlysimeters and un-leached beakers) in two soils (i.e. high and low C:N ratios) and in *row-2*, two soils when incubated in two incubation systems. Data tested are cumulative carbon mineralised relative to total soil organic carbon (Fig 3) over the 121-day incubation (g kg^-1^ OC). Cell values: Significance code based on *p-*values (‘‘1, ‘.’0.1, ‘*’0.05, ‘**’0.01, ‘***’0.001), *t-*value, p = *p*-value.
(DOCX)
