# Supplementary material for: Leaching of soils during laboratory incubations does not affect soil organic carbon mineralisation but solubilisation
Source: PLoS One. 2017 Apr 5;12(4):e0174725. doi: 10.1371/journal.pone.0174725 (PMC5381879; doi:10.1371/journal.pone.0174725)
Supplement: S1 Table — Data tested are soluble mineral nitrogen (NO3--N and NO2--N) relative to total soil organic carbon (Fig 1A and 1B) over the 121-day incubation (g kg-1 OC). SWEe-Nmin was measured in 1:5 soil water extracts (Fig 1A) and SWET-Nmin was calculated (Eq 1) for the total soil water extract as the sum of extracted and leached Nmin (Fig 1B). Cell values: Significance code based on p-values (‘‘1, ‘.’0.1, ‘*’0.05, ‘**’0.01, ‘***’0.001), t-value, p = p-value. (DOCX) [file pone.0174725.s001.docx]

**S1 Table -** Results of Student’s *t-*tests to compare two incubation systems (i.e. leached microlysimeters and un-leached beakers) in two soils (i.e. high and low C:N ratios). Data tested are soluble mineral nitrogen (NO_3_^‑^-N and NO_2_^‑^-N) relative to total soil organic carbon (Fig. 1 a and b) over the 121-day incubation (g kg^-1^ OC). SWE_e_-N_min ­_was measured in 1:5 soil water extracts (Fig. 1a) and SWE_T_-N_min_ was calculated (Eq. 1) for the total soil water extract as the sum of extracted and leached N_min_ (Fig. 1b). Cell values: Significance code based on *p-*values (‘’1, ‘.’0.1, ‘*’0.05, ‘**’0.01, ‘***’0.001), *t-*value, p = *p*-value.

|  | **4 days** | | **13 days** | | **30 days** | | **63 days** | | **121 days** | |
| --- | --- | --- | --- | --- | --- | --- | --- | --- | --- | --- |
|  | Low C:N | High C:N | Low C:N | High C:N | Low C:N | High C:N | Low C:N | High C:N | Low C:N | High C:N |
| **SWE_e_-N_min_** | 0.10675,  p = 0.9202 | 0.1105,  p = 0.9177 | 0.87383,  p = 0.4471 | 1.1197,  p = 0.339 | 2.0216,  p = 0.1204 | ******  6.7972,  p = 0.00344 | **.**  2.31,  p = 0.09617 | 0.89228,  p = 0.439 | *****  3.9307,  p = 0.02424 | -0.20571,  p = 0.8485 |
| **SWE_T_-N_min_** | 0.10675,  p = 0.9202 | 0.1105,  p = 0.9177 | 0.3103,  p = 0.7747 | 0.99032,  p = 0.3813 | 0.13553,  p = 0.8988 | -1.1377,  p = 0.3399 | -0.053909,  p = 0.9596 | -0.090526,  p = 0.9328 | 0.69646,  p = 0.5245 | -0.95545,  p = 0.3994 |
