# Supplementary material for: Leaching of soils during laboratory incubations does not affect soil organic carbon mineralisation but solubilisation
Source: PLoS One. 2017 Apr 5;12(4):e0174725. doi: 10.1371/journal.pone.0174725 (PMC5381879; doi:10.1371/journal.pone.0174725)
Supplement: S2 Table — Data tested are soluble organic carbon (OC) relative to total soil organic carbon (Fig 1A and 1B) over the 121-day incubation (g kg-1 OC). SWEe-OC was measured in 1:5 soil water extracts (Fig 2A) and SWET-OC was calculated (Eq 1) for the total soil water extract as the sum of extracted and leached OC (Fig 2B). Cell values: Significance code based on p-values (‘‘1, ‘.’0.1, ‘*’0.05, ‘**’0.01, ‘***’0.001), t-value, p = p-value. (DOCX) [file pone.0174725.s002.docx]

**S2 Table -** Results of Student’s *t-*tests to compare two incubation systems (i.e. leached microlysimeters and un-leached beakers) in two soils (i.e. high and low C:N ratios). Data tested are soluble organic carbon (OC) relative to total soil organic carbon (Fig. 1 a and b) over the 121-day incubation (g kg^-1^ OC). SWE_e_-OC _­_was measured in 1:5 soil water extracts (Fig. 2a) and SWE_T_-OC was calculated (Eq. 1) for the total soil water extract as the sum of extracted and leached OC (Fig. 2b). Cell values: Significance code based on *p-*values (‘’1, ‘.’0.1, ‘*’0.05, ‘**’0.01, ‘***’0.001), *t-*value, p = *p*-value.

|  | **4 days** | | **13 days** | | **30 days** | | **63 days** | | **121 days** | |
| --- | --- | --- | --- | --- | --- | --- | --- | --- | --- | --- |
|  | Low C:N | High C:N | Low C:N | High C:N | Low C:N | High C:N | Low C:N | High C:N | Low C:N | High C:N |
| **SWE_e_-OC** | -0.84006,  p = 0.459 | -0.17068,  p = 0.8728 | -0.80399,  p = 0.4875 | 1.3264,  p = 0.2554 | -0.090158,  p = 0.9326 | 0.31998,  p = 0.7665 | 1.3385,  p = 0.2518 | 0.4969,  p = 0.6627 | 0.71743,  p = 0.5165 | -0.27411,  p = 0.7979 |
| **SWE_T_-OC** | -0.84006,  p = 0.459 | -0.17068,  p = 0.8728 | -0.91404,  p = 0.437 | 0.925,  p = 0.4079 | -0.48406,  p = 0.6547 | *****  -3.5975,  p = 0.02671 | 0.62969,  p = 0.5632 | -1.0406,  p = 0.3949 | -0.16474,  p = 0.8783 | **.**  -2.481,  p = 0.07286 |
