# Supplementary material for: Leaching of soils during laboratory incubations does not affect soil organic carbon mineralisation but solubilisation
Source: PLoS One. 2017 Apr 5;12(4):e0174725. doi: 10.1371/journal.pone.0174725 (PMC5381879; doi:10.1371/journal.pone.0174725)
Supplement: S3 Table — Data tested are cumulative carbon mineralised relative to total soil organic carbon (Fig 3) over the 121-day incubation (g kg-1 OC). Cell values: Significance code based on p-values (‘‘1, ‘.’0.1, ‘*’0.05, ‘**’0.01, ‘***’0.001), t-value, p = p-value. (DOCX) [file pone.0174725.s003.docx]

**S3 Table -** Results of Student’s *t-*tests to compare: in *row-1*, two incubation systems (i.e. leached microlysimeters and un-leached beakers) in two soils (i.e. high and low C:N ratios) and in *row-2,* two soils when incubated in two incubation systems. Data tested are cumulative carbon mineralised relative to total soil organic carbon (Fig. 3) over the 121-day incubation (g kg^-1^ OC). Cell values: Significance code based on *p-*values (‘’1, ‘.’0.1, ‘*’0.05, ‘**’0.01, ‘***’0.001), *t-*value, p = *p*-value.

|  | **4 days** | | **13 days** | | **30 days** | | **63 days** | | **121 days** | |
| --- | --- | --- | --- | --- | --- | --- | --- | --- | --- | --- |
|  | Low C:N | High C:N | Low C:N | High C:N | Low C:N | High C:N | Low C:N | High C:N | Low C:N | High C:N |
| **Carbon mineralised** | -0.71057,  p = 0.5168 | 0.43337,  p = 0.6975 | -0.15603, p = 0.8846 | 0.28752,  p = 0.7916 | 0.00025531,  p = 0.9998 | -0.27999,  p = 0.8023 | -0.088176,  p = 0.9344 | -0.159,  p = 0.8833 | -0.24033,  p = 0.8234 | -0.97237,  p = 0.3925 |
|  | Beaker | Micro | Beaker | Micro | Beaker | Micro | Beaker | Micro | Beaker | Micro |
|  | 0.47945,  p = 0.6686 | **.**  2.4749,  p = 0.06918 | 0.48593,  p = 0.6528 | 0.86013,  p = 0.4589 | -1.0908,  p = 0.3378 | -1.1425,  p = 0.3658 | -0.54884,  p = 0.6128 | -0.56654,  p = 0.6133 | 0.67486,  p = 0.5423 | 0.30044,  p = 0.7882 |
